# Reconstructive management of the rare bilateral oral submucos fibrosis using nasolabial flap in comparison with free radial forearm flap - a randomised prospective trial

**DOI:** 10.1186/1750-1172-8-56

**Published:** 2013-04-08

**Authors:** Muhammad Faisal, Madiha Rana, Anjum Shaheen, Riaz Warraich, Horst Kokemueller, André Michael Eckardt, Nils-Claudius Gellrich, Majeed Rana

**Affiliations:** 1 Department of Oral and Maxillofacial Surgery, Nishtar Institute of Dentistry, Multan, Pakistan; 2 Department of Craniomaxillofacial Surgery, Hannover Medical School, Carl-Neuberg-Street 1, Hannover D-30625, Germany

**Keywords:** Oral sub mucous fibrosis, Nasolabial flap, Radial free forearm flap, Microvascular anastomosis

## Abstract

**Background:**

Oral sub mucous fibrosis is a rare chronic, progressive, pre malignant collagen disorder of oral mucosa in people of Asian descent characterized by trismus, blanching and stiffness of mucosa, burning sensation in mouth and hypomobility of soft palate and tongue with loss of gustatory sensation. Betel nut chewing is the most common etiological agent. Surgery remains the main stay in severe cases and aims at release of fibrotic bands and resurfacing the raw areas with different options. Reconstruction can be done by using nasolabial flap or radial free forearm flap. The purpose of this study was to compare the mouth opening after the reconstruction with either nasolabial flap or radial free forearm flap.

**Methods:**

This study was carried out on fifty (50) patients with oral sub mucous fibrosis. Twenty five (25) of these were reconstructed by nasolabial flap and twenty five (25) were reconstructed by radial free forearm flap. At different intervals of their post-operative visits, they were evaluated for the interincisal distance and the difference between the two groups was assessed.

**Results:**

Average increase in interincisal distance was greater in patients reconstructed with radial free forearm flap compared with patient reconstructed by nasolabial flap i.e. 18.96 mm and 15.16 mm respectively with ‘P’ value > 0.05.

**Conclusion:**

Based on this study radial forearm free flap is a superior method compared to transposition of nasolabial flap to cover the surgical wound of oral submucous fibrosis.

## Background

Oral submucous fibrosis (OSMF) is a rare chronic, debilitating premalignant condition characterised by juxtaepithelial fibrosis of the oral cavity associated with an inflammatory reaction followed by fibroelastic change of the lamina propria and epithelial atrophy that leads to stiffness of the oral mucosa and causes trismus and an inability to eat. It is most common in South East Asia. It is reported that incidence of oral submucous fibrosis was more in women among the cases [1-3]. Across the world 2.5 million people are affected [4]. Evidences based on epidemiological studies, large cross-sectional surveys and interventional studies have shown that areca-nut is the main aetiological factor in OSMF. Areca nut is one of the most widely used psychoactive substances with several hundred million users worldwide, predominantly in southern Asia. The habit of chewing betel quid, containing fresh, dried or cured areca nut, catechu with or without tobacco, slaked lime and flavoring ingredients wrapped in betel leaf is widespread in India, Pakistan, Bangladesh and Sri Lanka and in immigrant populations from these regions. An estimate from 1996 indicated that globally, about 2.5 million people have Oral submucous fibrosis but studies have found that over 5 million people are affected in India alone (0.5% of the Indian population) [1,5]. Experimentally Arecoline, a derivative of areca can induce fibroblast proliferation and collagen synthesis. There is a significant association between Areca nut consumption and sub mucous fibrosis [6]. Other factors are genetic and immunologic processes, ingestion of chilies and nutritional deficiency such as iron vitamin B_12_ and folic acid [2].

The most obvious clinical signs include blanched, opaque oral mucosa with palpable fibrous bands. Furthermore, the overlying epithelium may become dysplastic and malignant. Restricted mouth opening interferes with examination of the oral mucosa, and makes early diagnosis of cancer a daunting task [7]. Occasionally soft palate, lips, pharynx and esophagus are involved [2]. Oral submucous fibrosis is essentially a disease of collagen metabolism with marked focus on increased synthesis, or reduced degradation, of collagen, as possible mechanisms in the development of the disease; there are changes in the normal collagen metabolism at different stages. Areca-nut contains alkaloids, flavonoids, and copper, which all interfere with homeostasis of the extracellular matrix. Four alkaloids – arecoline (most potent), arecaidine, guvacine, and guvacoline – are known to stimulate fibroblasts to produce collagen Flavonoids (tannins and catechins) inhibit collagenase, stabilise the collagen fibrils, and render them resistant to degradation by collagenase [6,8]. Oral submucous fibrosis has a malignant transformation rate of 7–30%. Areca nut which is a group one carcinogen has synergestic effect with tobacco. Recently, a loss of heterozygosity in 23 “hotspot” loci which alter genes that control the cell cycle has been recognised as an important molecular marker for malignancy in Oral submucous fibrosis [9,10].

Prognosis of submucous fibrosis is poor and in patients with oral submucous fibrosis, the risk of developing oral carcinoma is 7.6% over a 10-year period [11]. If the palatal and paratubal muscles are involved in patients with oral sub-mucous fibrosis, conductive hearing loss may occur because of functional stenosis of the Eustachian tube [12]. No treatment is effective in patients with oral sub-mucous fibrosis, and the condition is irreversible [13]. Recent reports claim improvement of the condition if the habit is discontinued following diagnosis at an early stage [14]. Medical treatment is conservative and is applied where surgery is contraindicated. It is only a palliative measure [2]. Surgical treatment involves simple excision of fibrotic bands and reconstruction. Simple excision of fibrotic bands causes contraction of tissue and contracture. Reconstruction can be done by split thickness skin graft [6], buccal pad of fat [15], nasolabial flap [16] or radial free forearm flap [17].

The purpose of this study was to compare the immediate and late outcomes of two reconstructive techniques with nasolabial flap and radial free forearm flap, determine the efficacy of two reconstructive options and to measure the mouth opening level by measuring interincisal distance after these two procedures.

## Material and methods

Approval for the study was obtained from the relevant ethics committee at the Nishtar Institute of Dentistry (NID/2003-066-161). Study subjects were enrolled in a clinical protocol reviewed and approved by the institutional cancer board. In addition, positive written consent was obtained from each subject who participated in the study.

**Figure 1:**
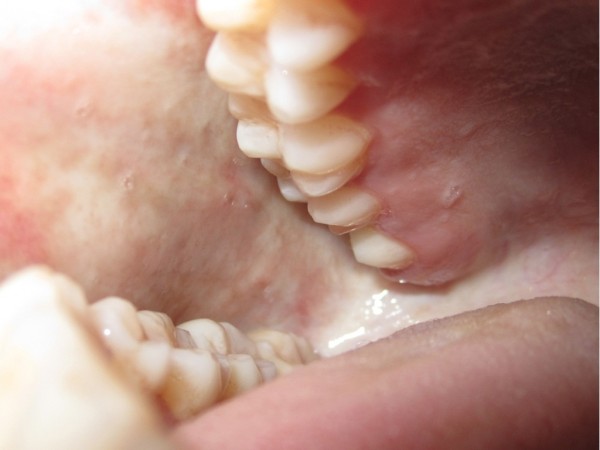
Buccal view of a patient with a histologically confirmed oral submucous fibrosis.

**Figure 2:**
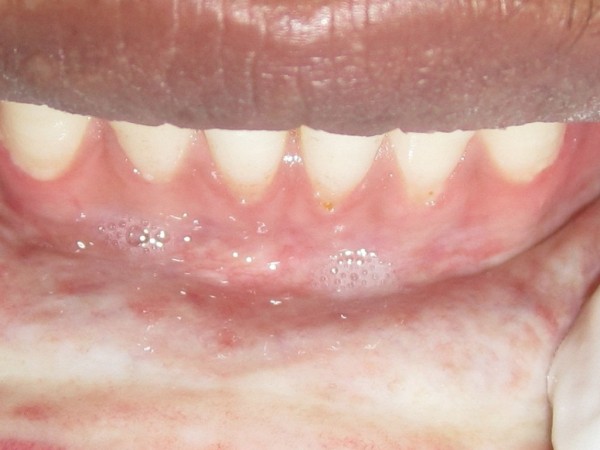
Frontal intraoral view shows typically precancerous condition with increased prevalence in the Indian subcontinent.

A total of 50 adults without sexual discrimination diagnosed clinically and histopathologically for oral submucous fibrosis, were prospectively and observer blind enrolled. The division in two groups occurred randomly. All patients were diagnosed with bilateral oral submucous fibrosis of buccal mucosa (Figures 1 &2). Exclusion criteria were patients having oral submucous fibrosis extending into pharynx, soft palate, oesophagus or paratubal muscles, malignant change in oral sub mucous fibrosis on biopsy, patients having diabetes mellitus, hypercholesterolemia, arteriosclerosis, blood coagulopathies, collagen vascular disorders and other vascular disorders, previously irradiated patients, intravenous drug abuse.

## Consort flow diagram

At the time of presentation 70 patients were assessed for eligibility to be included in the study. Out of these 14% of the patients (n = 10) were not included in the study as 8% patients (n = 6) did not meet the inclusion criteria while 5% (n = 4) did not want to participate in the study. A total of 60 patients were randomly allocated in two groups with 30 patients allocated in each group for intervention. In the group treated by nasolabial flap 100% patients (n = 30) received the selected intervention. In the group operated with radial forearm free flap 100% patients (n = 30) received the selected intervention. Among the 30 patients who were reconstructed using nasolabial flap 3% (n = 1) was lost to follow-up as these patients come from far areas and could not travel due to economic or personal reasons. 6% (n = 2) of the patient died of cardiac failure. While the 30 patients who were managed using radial forearm free flap, 6% (n = 2) were lost to follow-up.

27 patients who received treatment using nasolabial flap in group 1 were available for follow-up, 2 of them had their data lost during the data analysis procedure. So the total number of patients who were analyzed for nasolabial flap was 25.

**Figure 3:**
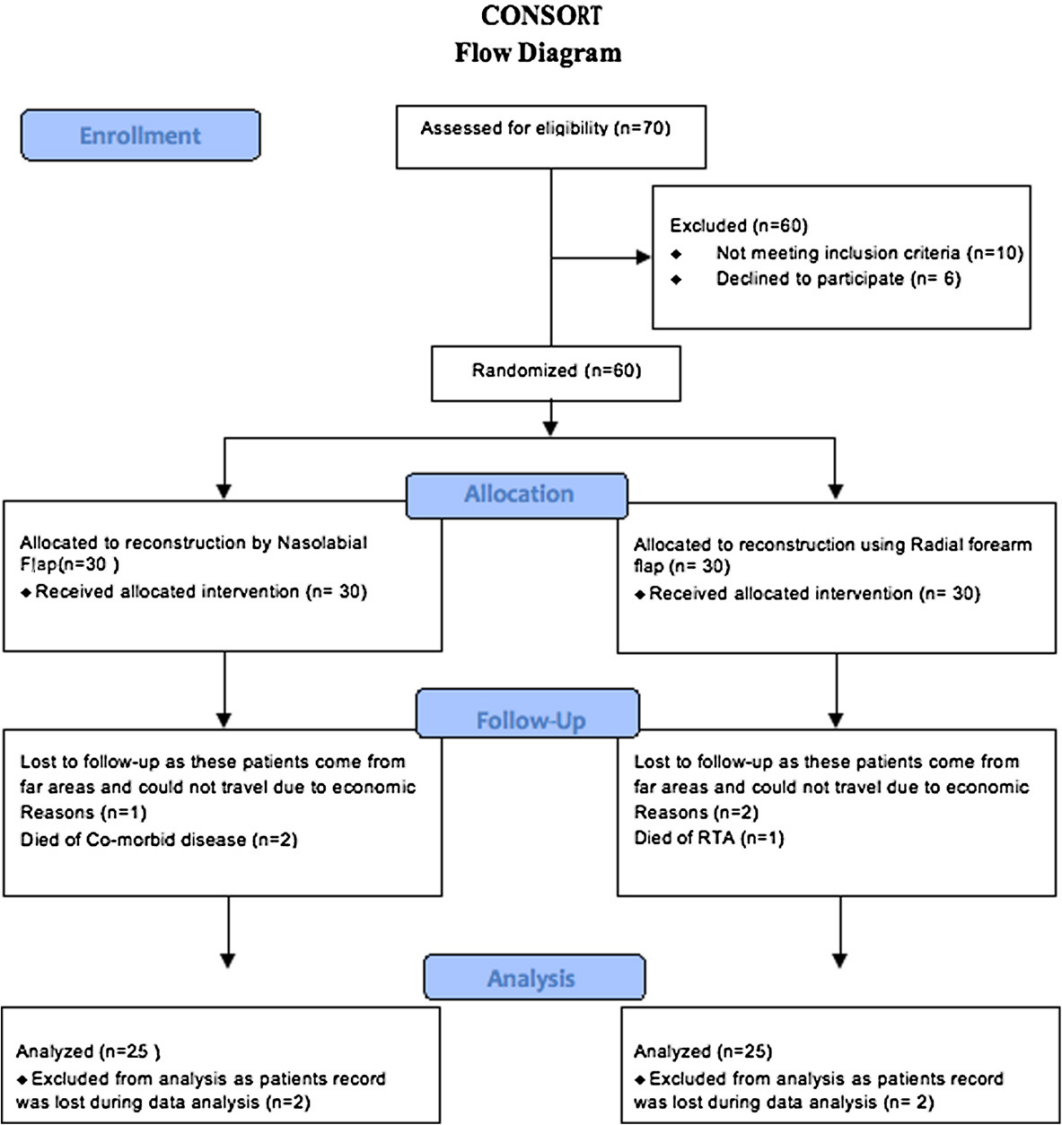
Demonstrates the consort flow diagram, which shows the enrollment, allocation, follow-up and data analysis.

The 28 patients who were managed using radial forearm free flap in group 2 were available for follow up, 2 of them had their data lost during the data analysis procedure, while 1 died of road traffic accident. So the total number of patients who were analyzed for RFFF was 25 (Figure 3).

## Randomization

Randomization was done using computer based software “RANDI2”. The software was used to generate serial numbers 1–70 into two groups randomly and those patients who fulfilled the inclusion criteria were allocated serial numbers according to date and sequence of admission to hospital. The person responsible for conducting the measurements at the time of assessment of variables was blindfolded regarding the type of procedure that was conducted. Data was collected on a specially designed Performa, taking account of habits, risk factors of oral sub mucous fibrosis along with the treatment options offered and their outcome postoperatively. The patients were recalled for follow up after every 15 days for six months average.

## Postoperative inter-incisal distance

Preoperatively mouth opening was measured by measuring interincisal distance (IID) in mm while the patient’s mouth is fully opened. Early post operative interincisal distance IID that was measured within first to third post operative day was also measured in mm. Late postoperative interincisal distance IID, measured at least one month after operation that was also measured in mm. Net increase in mouth opening that was the difference between post operative IID and preoperative IID. It was also measured in mm. Shrinkage of flap that was a difference between early post operative IID and late post operative IID and it was also measured in mm.

## Viability of flap

Viability of flap was checked by Doppler pencil probe or prick test after every 1–2 hours in first 72 hours after surgery. Color of flap was analyzed visually.

## Wound dehiscence

Wound was assessed postoperatively by the size and surface area of the wound. It may be partial or complete breakdown.

## Donor site morbidities

Donor site morbidities are assessed by way of suture breakdown, wound dehiscence and infection at the site from where flap was harvested.

## Patient satisfaction of surgical treatment

All patients were given a questionnaire before discharge of hospital. Patients were asked based on the subjective perception of the comfort and satisfactory concerning the post-operative mouth opening. The data were graded in a scale ranging from 1 to 4, while 1 was set as very satisfied and 4 not satisfied.

## Statistical analysis

The variables of this study were presented as proportions. The proportions in two groups were compared using the chi-square test with one degree of freedom and at an alpha level at 0.05. This was done using SPSS 14.0 (SPSS Inc., Chicago, IL, USA) on a computer. The data based on interincisal distance was assessed for quantitative measures and was tested by “t” test of significance. Other variables included age, gender, etiology of oral sub mucous fibrosis, infection, and flap necrosis, function of donor site, donor site difficulties, and wound healing difficulties. Such type of data was analyzed by chi-square test (fisher’s exact test).

## Results

### Baseline characteristics

50 patients requiring surgical intervention for oral submucous fibrosis were enrolled in this clinical comparative study. Patients were randomly allocated in two groups with group A (n = 25) which underwent reconstruction using nasolabial flap and group B (n = 25) in which patients were operated with the use of radial forearm free flap. The clinical and demographic characteristics of patients in both groups are shown in Table 1.

**Table 1:** Baseline characteristics of patients

	**Nasolabial flap**	**Radial flap**	***P *value**
Gender female – no./total no. (%)	9/25 (33)	4/25 (14)	0.001
Age (years) ± SD	44.6 ± 8.6	41.8 ± 9.8	0.295
Operation duration (minutes) ± SD	117.6 ± 39.6	232.8 ± 39.0	0.001
Hospitalization duration (days) ± SD	2.6 ± 0.6	5.6 ± 1.1	0.001
Preoperative mouth opening (mm) ± SD	6.6 ± 2.2	6.2 ± 2.4	0.543
Postoperative mouth opening (mm) ± SD	25.4 ± 6.3	27.2 ± 3.76	0.227
Increase in Mouth opening (mm) ± SD	15.2 ± 8.5	18.9 ± 6.16	0.085
1 Month Post-Op mouth opening (mm) ± SD	27.1 ± 8.6	28.8 ± 9.6	0.513

Buccal mucosa was involved in all of the cases. The most common etiological factor is found to be Betel quid (88%) followed by betel nut (10%). None of the patient had nutritional deficiency Table 2.

**Table 2:** Etiology

***Factors***	***n***	***Average duration***	***Smoking***
Betel quid chewing	44	20	29
Betel nut chewing	5	15	1
Betel quid and betel nut chewing	1	10	0

## Postoperative inter-incisal distance

Following surgery, both groups has shown no significant improvement in mouth opening but results were better in group B where net increase in mouth opening is 18.96 ± 6.16 as compared to group A which is 15.16 ± 8.5 (Table 1).

**Figure 4:**
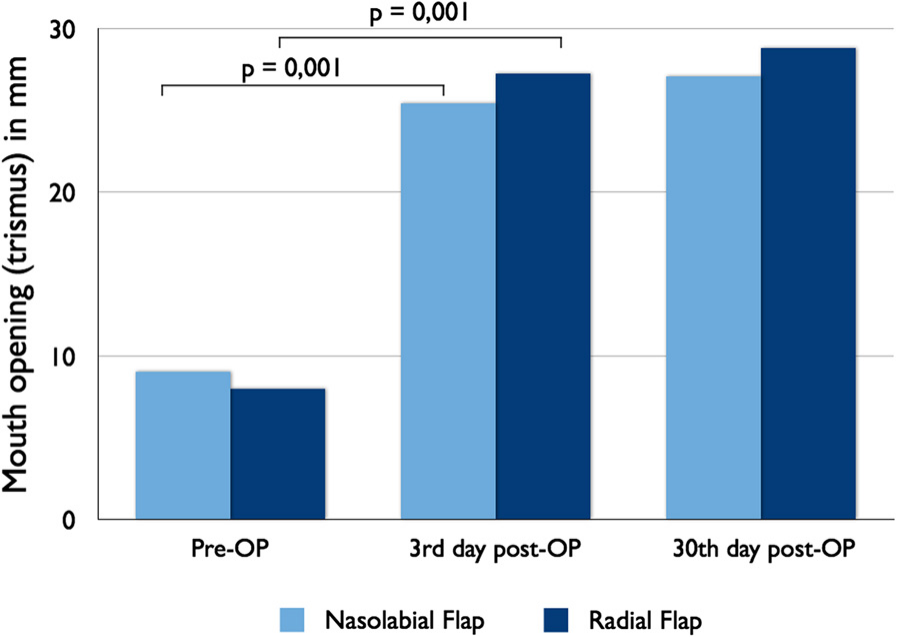
**Pre-operative and post-operative mouth opening values did differ significantly in both groups.** 3rd day after treatment of bilateral submucose fibrosis mouth opening climbed to healthy vales and no differences were observed comparing both groups and in comparing to both procedures.

The difference between early postoperative interincisal distance and late postoperative interincisal distance shows the shrinkage of flap but still mouth opening is clinically better in group B (28.84 ± 9.66 mm vs 27.08 ± 8.6 mm) (Figure 4).

## Flap necrosis and viability of flap

**Figure 5:**
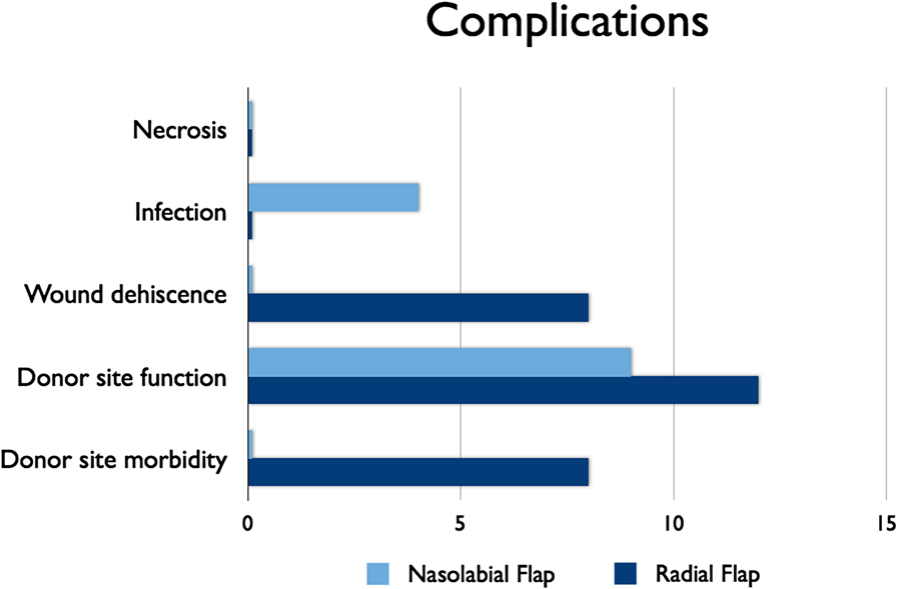
Shows the complication rates comparing both procedures regarding flap necrosis, infection, wound dehiscence, donor site function and donor site morbidity.

No patient in either group A or B experienced necrosis of flap. Viability of flaps were normal (Figure 5).

### Wound dehiscence

Wound healing at the donor and recipient site in group A is better than group B where wound dehiscence was present in 2 (8%) patients at the donor site (Figure 5).

### Infection

In group A, 1 (4%) of the patient got infection of the flap while in group B there is no reported incidence of infection (Figure 5).

### Donor site function

In group A, all the patients had good function of donor site. All the facial expression (smile, laughing, talking etc.) were excellent.

In group B, at the scar at the donor site, five items (pigmentation, scar width, depression, wrist mobility, and sensory abnormalities) were evaluated. Depression and pigmentation were often observed, but patient dissatisfaction was slight. Mobility of hand of the donor site was limited in one patient. So the result was good in 96% of patients. On the questionnaire, 2 patients (8%) reported slightly impaired function, 1 (4%) reported numbness, 5 (25%) reported itching, 2 (8%) reported cold intolerance, 5 (25%) reported bad cosmetic appearance.

Sensory deficit at donor site in group B was evaluated. 3 patients out of 25 (12%) had loss of all sensations of donor site hand and 5 (25%) reported bad cosmetic appearance (Figure 5).

### Patient satisfaction

**Figure 6:**
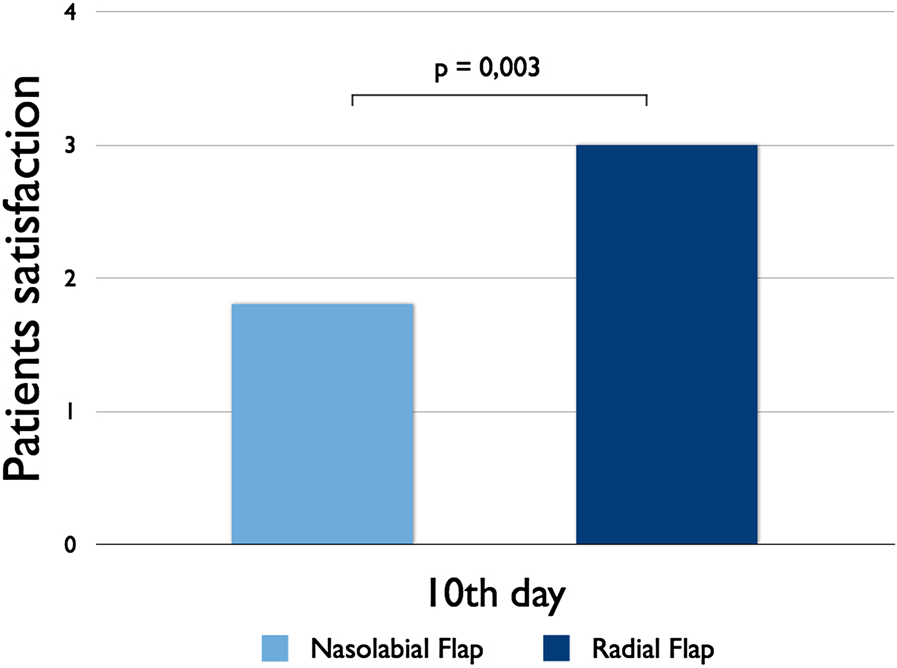
The overall satisfaction was significantly lower of patients receiving nasolabial flap compared to patients receiving radial forearm flap.

Regarding the patient´s satisfaction, which was assessed at 10^th^ day after surgery, a statistically significant difference between group I and group II could be detected (nasolabial: 1.8 ± 0.2, radial forearm: 3.0 ± 0.3, *p* = 0.003) (Figure 6).

## Discussion

In this study, group A patients included excision of the lesion and reconstruction with nasolabial flap and group B included excision and reconstruction with radial free forearm flap. Functional outcomes as well as success rate of flaps and morbidity of procedure were measured. There was significant achievement in mouth opening after the two reconstructive procedures. This finding is consistent with the previous studies that also showed that mouth opening is greatly improved after reconstruction with either nasolabial flap [18] or radial free forearm flap [1,17].

While evaluating the comparison of improvement in mouth opening after two reconstructive procedures, it was observed that mouth opening was improved more in group B (18.96 mm in group B and 15.16 mm in group A). This difference in improvement of mouth opening is not so significant owing to small sample size. Further study in this subject is recommended.

Flap necrosis did not occur in any group in our study which is better than the previous studies [19-21]. Both the nasolabial flap and the radial free forearm flap had excellent vascularity. Nasolabial flap is an arterialized pedicled flap with an axial blood supply. It also takes its vascularity from the recipient bed within two to three weeks. Radial free forearm flap is a free flap in which microvascular anastomosis of radial artery has been done with the recipient vessel, usually the facial artery. So the vascularity of this flap is much higher.

This comparison shows that while using radial free forearm flap, there may be chances of complications and greater post operative care is needed for better outcome.

Regarding wound dehiscence, result was 96% good in group A and 100% good in group B at recipient site but at donor site, result was 100% good in group A and 92% good in group B, as wound dehiscence was observed in two patients. So the condition of wound was better at recipient site in group B and at donor site in group A.

**Figure 7:**
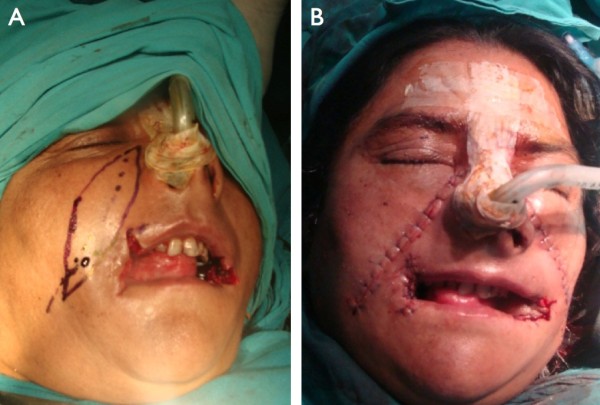
(A) Intraoperative view of a patient with bilateral submocose fibrosis, marked before harvesting the nasolabial flap (B) Direct postoperative view after reconstruction with nasolabial flap.

These findings are due to the fact that donor site is closed primarily while using the nasolabial flap. During this primary closure the margins of the wound area are released from the underlying tissue in order to allow approximation of both wound edges. But this primary closure may cause tension on the stitches and the wound may dehisce. This may be true in young patients but in old aged, there is lesser amount of fat and greater amount of loose areolar tissue in subcutaneous tissue, there is good approximation of both wound margins. So there is no tension on stitches and the chances of wound dehiscence are less. In our study it never happened in both young and old age groups while using nasolabial flap (Figure 7A & B).

But in case of radial free forearm flap, there is absent of subcutaneous fat and lesser amount of loose areolar tissue. So the undermining and approximation of wound edges is not always possible. We can do it in only that cases in which defect size is too small. But again tension on stitches may occur even the defect size is too small and stitches may break causing dehiscence of wound at that places. And this thing happened in one of my cases. When the defect size is large, we may close it using skin graft from the thigh area of the patients. But again there are chances that skin graft may get infected or it may not be taken up by the recipient site. Infection of the graft occurred in one case but that was managed by proper antibiotics and after washing the grafted area with 0.9% normal saline repeatedly.

In the study group of radial free forearm flap, one patient was presented with excessive hair growth at the flap during follow up period. As this is a rare finding but is also presented by Endo [22] in one of his case.

As regarding the function of donor site, it was 100% excellent in group A and 92% good in group B as one patient experienced limited mobility of donor site hand. This patient did not follow the instructions about the hand exercises properly. The reason is that the nerve supplying the wrist muscles may be severed along with flap. Three patients (12%) had loss of all sensations of donor site hand.

As the donor site problems is concerned, the work of Wei [23] done on fifteen patients reconstructed with bilateral radial free forearm flap after surgical release of sub mucous fibrosis, he found gangrene of finger tips in one patient who was heavy smoker.

While studying the work of Chen [24], he presents his clinical experience with head and neck reconstruction using the FRFF and the morbidity of the donor sites. Of the 38 FRFFs, 35 FRFFs were performed successfully. The survival rate of FRFF was 92% which is well in accordance to our study. Donor site complications included partial loss of skin graft in 4 donor sites (11%), abnormal sensations in 10 (26%), poor appearance in 3 (8%), and reduced grip strength in 4 (11%).

In one study of Kerawala [25], fifty patients undergoing radial forearm free flap reconstruction of head and neck defects were examined to find out the extent of sensory defect at the donor site. Of the 50 patients 38 (76%) were aware of some sensory loss over the radial distribution in the donor hand which is a little high as compared to our results.

The results concerning morbidity of the fasciocutaneous radial forearm flap donor site of 20 patients presented by Kropfl [26] showed same concerns of dysaesthetic areas in patients on the radial border of the donor site and reduced sensation of the radial nerve was present in four patients.

Swanson work done on thirty-five consecutive patients treated with the radial forearm flap showed higher percentage of donor-site complications included partial loss of the skin graft with tendon exposure in, an unsatisfactory appearance in, and one case of radial fracture.

Therefore, we believe that, because of the reliability, functional characteristics, and low donor site morbidity, the FRFF is a useful and versatile flap for reconstruction of head and neck defects.

Infection did not occur in any case of group B. Reason is that while using radial free forearm flap micro vascular anastomosis of radial artery has been done at the recipient site with the facial artery that provides excellent vascularity of the flap. This excellent blood supply of the flap combats with the infection. The work of other authors also proves that there are minimum chances of infection in radial free forearm flap reconstructed in this location. Nasolabial flap has also good blood supply so infection is not a major problem. Only one flap got infected. Previous studies also showed that chances of infection in this flap are less [20,21].

Furthermore, repeated debulking of the subcutaneous fat and small vestibuloplasties were necessary in both group of patients. Microsurgically revascularized tissue transfer has won a superior position in complex reconstruction in the head and neck field over the last 20 years. Jones [27], Chen et al. [28] declared this flap to be “ideal for intraoral use, providing thin, hairless skin with a long, large-caliber vascular pedicle.” In spite of the fact that the radial forearm flaps were reported to be comparably thin, debulking always became necessary [29]. The use of radial free forearm flap is aesthetically pleasing procedure as it produces no scar on face. Change of appearance in group A is also minimal and very acceptable to the patients. As the donor site of nasolabial flap area is closed primarily, the overlying skin is stretched that removes the wrinkles on the face. This effect is aesthetically pleasing to the patient. This effect is similar to the face lift procedure or rhytidectomy. But it produces a scar mark on the face. This scar mark is acceptable to patients as it is hidden in the nasolabial fold that is a natural skin crease. But some aesthetic conscious patients are not satisfied with this appearance. The patients in whom reconstruction with nasolabial flap was done only unilaterally did not have a problem of facial asymmetry as greater undermining was done to mask the effect. This is not a problem in reconstructing radial free forearm flap [16] Sidebottom [30] in 2000 filled a questionnaire asked from the patients reconstructed with radial free forearm flap, during follow up visits. He reported, no patient was dissatisfied with his aesthetics.

The results of 13 single-stage reconstructions of large and complex defects in the head and neck by using eight free radial forearm flaps were analyzed by Natschev [31]. The results proved to be better functionally and aesthetically.

As regarding the shrinkage of the flap, it was minimal in group B. Rather mouth opening was improved with passage of time in both groups (24% and 32% of patients in respectively group A and B). In group A, shrinkage of flap was more marked (13 mm) in two patients who did not follow the stick exercises after reconstruction. So the shrinkage of flap occurred in those patients. This problem associated with nasolabial flap has been described in other studies. Minor shrinkage of about 1 – 2 mm occurred in patients who follow the stick exercise but not as strictly as they should in order to achieve maximum results. The increase in interincisal distance of about 1 – 6 mm in late post operative visits is due to the facts that patient is reluctant to open his mouth properly due to pain and swelling caused by the surgical trauma during the 1^st^ to third post operative stay in the ward. There might be a fear in his mind that if he/she will open his/her mouth, stitches will break down. When these patients follow the stick exercises strictly, their mouth opening is improved.

While comparing the results of my study population with the work of Celik [17] done on fifteen patients with bilateral radial free forearm flaps after surgical release of oral sub mucous fibrosis, average shrinkage of about 5 mm was noticed in patients in late post operative period. Stick exercise of jaw should be started from the 1^st^ post operative day. Those patients who follow it strictly from the very 1^st^ day has improvement in mouth opening at the first post operative day that is maintained in the follow up visits.

## Conclusion

This study shows that there was significant achievement of mouth opening after the excision of fibrous bands along with reconstruction with either nasolabial flap or with radial free forearm flap. While comparing these two procedures, results show that while reconstructing with radial free forearm flap, improvement in mouth opening is by trend better. Although, the difference in improvement in mouth opening by the two procedures was not significant. But there is a tendency of a better improvement of mouth opening with radial forearm flap. There are also comparatively less chances of infection, necrosis, wound healing problems and shrinkage of flap in reconstruction with radial free forearm flap but the problems associated with donor site are more with the use of this flap.

## Competing interests

The authors declare that they have no competing interests.

## Authors’ contributions

MF, MAR, AS, RW, HK, AME, NCG and MR conceived of the study and participated in its design and coordination. MF and MR made substantial contributions to conception and design of the manuscript as well as data acquisition. AS, RW, HK NCG and AME were involved in revising the manuscript. MR and MF drafted the manuscript. MAR made the statistical analysis. All authors read and approved the final manuscript.
