# Correction: Reconstructive management of the rare bilateral oral submucos fibrosis using nasolabial flap in comparison with free radial forearm flap - a randomised prospective trial

**DOI:** 10.1186/1750-1172-8-86

**Published:** 2013-06-14

**Authors:** Muhammad Faisal, Madiha Rana, Anjum Shaheen, Riaz Warraich, Horst Kokemueller, André Michael Eckardt, Nils-Claudius Gellrich, Majeed Rana

**Affiliations:** 1 Department of Oral and Maxillofacial Surgery, Nishtar Institute of Dentistry, Multan, Pakistan; 2 Department of Craniomaxillofacial Surgery, Hannover Medical School, Carl-Neuberg-Street 1, Hannover D-30625, Germany

## Abstract

**Background:**

Oral sub mucous fibrosis is a rare chronic, progressive, pre malignant collagen disorder of oral mucosa in people of Asian descent characterized by trismus, blanching and stiffness of mucosa, burning sensation in mouth and hypomobility of soft palate and tongue with loss of gustatory sensation. Betel nut chewing is the most common etiological agent. Surgery remains the main stay in severe cases and aims at release of fibrotic bands and resurfacing the raw areas with different options. Reconstruction can be done by using nasolabial flap or radial free forearm flap. The purpose of this study was to compare the mouth opening after the reconstruction with either nasolabial flap or radial free forearm flap.

**Methods:**

This study was carried out on fifty (50) patients with oral sub mucous fibrosis. Twenty five (25) of these were reconstructed by nasolabial flap and twenty five (25) were reconstructed by radial free forearm flap. At different intervals of their post-operative visits, they were evaluated for the interincisal distance and the difference between the two groups was assessed.

**Results:**

Average increase in interincisal distance was greater in patients reconstructed with radial free forearm flap compared with patient reconstructed by nasolabial flap i.e. 18.96 mm and 15.16 mm respectively with ‘P’ value > 0.05.

**Conclusions:**

Based on the results of this study, there was no significant difference in mouth opening after reconstruction with radial forearm free flap compared to nasolabial flap.

## Correction

Since publication of this article [1] it has come to our attention that we had overstated our conclusions in the abstract. There was no significant difference in mouth opening between the two techniques. We have provided a revised abstract below.

## References

[B1] Faisal M Rana M Shaheen A Warraich R Kokemueller H Eckardt AM Gellrich NC Rana M Reconstructive management of the rare bilateral oral submucos fibrosis using nasolabial flap in comparison with free radial forearm flap - a randomised prospective trial Orphanet Journal of Rare Diseases 2013 8 56 10.1186/1750-1172-8-56 23915701 PMC3717039

